# Real-World Outcomes of Glucose Sensor Use in Type 1 Diabetes—Findings from a Large UK Centre

**DOI:** 10.3390/bios11110457

**Published:** 2021-11-15

**Authors:** Kyuhan Lee, Shakthi Gunasinghe, Alyson Chapman, Lynne A. Findlow, Jody Hyland, Sheetal Ohol, Andrea Urwin, Martin K. Rutter, Jonathan Schofield, Hood Thabit, Lalantha Leelarathna

**Affiliations:** 1 Medical School, University of Manchester, Manchester M13 9PL, UK; kyuhan.lee@student.manchester.ac.uk (K.L.); shakthi.gunasinghe@student.manchester.ac.uk (S.G.); 2 Manchester Academic Health Science Centre, Diabetes, Endocrinology & Metabolism Centre, Manchester University NHS Foundation Trust, Manchester M13 9WL, UK; alyson.chapman@mft.nhs.uk (A.C.); lynneann.findlow@mft.nhs.uk (L.A.F.); jody.hyland@mft.nhs.uk (J.H.); sheetal.ohol@mft.nhs.uk (S.O.); andrea.urwin@mft.nhs.uk (A.U.); martin.rutter@mft.nhs.uk (M.K.R.); jonathan.schofield@mft.nhs.uk (J.S.); hood.thabit@mft.nhs.uk (H.T.); 3 Division of Diabetes, Endocrinology & Gastroenterology, Faculty of Biology, Medicine and Health, University of Manchester, Manchester M13 9PL, UK

**Keywords:** type 1 diabetes, flash glucose monitoring, continuous glucose monitoring, Freestyle Libre, Dexcom G6

## Abstract

Flash glucose monitoring (FGM) and real-time continuous glucose monitoring (RT-CGM) are increasingly used in clinical practice, with improvements in HbA1c and time in range (TIR) reported in clinical studies. We aimed to evaluate the impact of FGM and RT-CGM use on glycaemic outcomes in adults with type 1 diabetes (T1DM) under routine clinical care. We performed a retrospective data analysis from electronic outpatient records and proprietary web-based glucose monitoring platforms. We measured HbA1c (pre-sensor vs. on-sensor data) and sensor-based outcomes from the previous three months as per the international consensus on RT-CGM reporting guidelines. Amongst the 789 adults with T1DM, HbA1c level decreased from 61.0 (54.0, 71.0) mmol/mol to 57 (49, 65.8) mmol/mol in 561 people using FGM, and from 60.0 (50.0, 70.0) mmol/mol to 58.8 (50.3, 66.8) mmol/mol in 198 using RT-CGM (*p* < 0.001 for both). We found that 23% of FGM users and 32% of RT-CGM users achieved a time-in-range (TIR) (3.9 to 10 mmol/L) of >70%. For time-below-range (TBR) < 4 mmol/L, 70% of RT-CGM users and 58% of FGM users met international recommendations of <4%. Our data add to the growing body of evidence supporting the use of FGM and RT-CGM in T1DM.

## 1. Introduction

Type 1 diabetes (T1DM) is a chronic autoimmune condition due to the destruction of insulin-producing beta cells in the pancreas [1]. Within the UK, it is estimated that approximately 29,000 children and 400,000 adults live with T1DM, with the prevalence increasing by 4% each year [2]

People with T1DM need to administer and self-adjust exogenous insulin to achieve normoglycaemia and minimise the risk of micro and macrovascular complications. Information on past, present, and predicted glucose levels are critical for effective self-adjustment of insulin doses, making it a fundamental foundation of modern T1DM self-management [3]. In the UK, less than one-third of adults with type 1 diabetes achieve the recommended HbA1c level < 59 mmol/mol (7.5%) [4]. The pain and inconvenience of conventional fingerstick capillary blood glucose testing remains a barrier to achieving optimal glucose control and is associated with low quality of life, poor treatment satisfaction and suboptimal adherence [5].

The present generation of sensor-based glucose monitoring devices provide a minimally invasive method to measure real-time interstitial fluid glucose levels [6]. Flash glucose monitoring (FGM), more specifically the Freestyle Libre system (FSL), was first introduced in 2014 in Europe. It consists of a 2-week, externally worn glucose sensor that displays present, 8-h historical, and trend glucose data when physically scanned by the user using a nearfield scanner [7]. In contrast to Flash glucose monitoring, real-time continuous glucose monitoring systems (RT-CGM) continuously display current glucose and trend information. In addition, real-time glucose monitors are also equipped with alerts for hypo and hyperglycaemia, including impending hypoglycaemia. Earlier generations of RT-CGM systems, such as Dexcom G5 required regular calibration with fingerstick glucose levels due to drift in sensor sensitivity. In contrast, Dexcom G6 is a factory-calibrated RT-CGM system that does not require fingerstick calibrations, and each sensor can work for up to 10 days [8,9]. In addition to providing glucose information, these systems support behavioural modification, as users will often respond to glucose measurements by adjusting insulin delivery, modifying eating habits, and exercise management.

Both FSL and Dexcom systems are available in the UK for people living with type 1 diabetes meeting specific NHS funding criteria (outlined in the Supplementary Table S1). Due to the relatively higher cost, the use of the Dexcom system is restricted to those with impaired awareness of hypoglycaemia, severe hypoglycaemia, or those with significant fear of hypoglycaemia.

The current glycaemic goal in non-pregnant adults with T1DM is to have a percentage of time spent in the target glucose range (3.9 to 10 mmol/L) over 70% while minimising the burden of hypoglycaemia (% time spent below 3.9 mmol/L and <3.0 mmol/L, less than 4% and 1% respectively, to minimise the risk of development of long-term diabetes-related complications [10,11].

The objective of this study was to assess the impact of starting FSL and Dexcom RT-CGM systems in T1DM adults under routine clinical care on glycaemic outcomes, as measured by HbA1c level and sensor-based metrics.

## 2. Materials and Methods

This was a retrospective, observational, single-centre service evaluation in a large UK teaching hospital. As a service evaluation, no ethical approval was required. Data were collected from electronic outpatient records and manufacturers’ proprietary web-based glucose monitoring platforms (Libreview; Abbott Diabetes Care; Oxon, UK and Dexcom Clarity; Dexcom Inc, San Diego, CA, USA). Patients had provided online informed consent for their data to be remotely linked and shared with the diabetes clinic staff. Data collection was undertaken in April and May 2021.

Patients were eligible for inclusion if they met the following criteria: history of T1DM, on multiple daily injections (MDI) or insulin pump therapy and using Freestyle Libre flash glucose monitoring (FSL) (First Generation) (Abbott Diabetes Care; Oxon, UK) or Dexcom (G5 or G6 versions) Dexcom Inc, San Diego, CA, USA). continuous glucose monitoring started before 31 December 2020. We calculated the mean baseline HbA1c from measurements taken in the 12 months before starting the device. Similarly, post-sensor start HbA1c was taken as the average of measurements taken up to 12 months after starting the device. If only one HbA1c level was available that value was used in calculations. We analysed the change in HbA1c according to three categories of baseline HbA1c: <59, 59–69 and >69 mmol/mol.

In keeping with international consensus on CGM reporting guidelines [10], we analysed the following glycaemic metrics of each patient: % time in glucose range (3.9–10.0 mmol/L) (TIR), % time below range (<3.9 mmol/L), % time above range (>10.0 mmol/L), coefficient of variation (CV%) and Glucose Management Index (GMI) for the three months prior to the time of data collection (April/May 2021). Adherence to sensor use was evaluated by assessing % of sensor data available for FSL and Dexcom continuous glucose monitors. All Dexcom users at the time of sensor data analysis were using Dexcom G6 sensor. We estimate that more than 95% of FSL users were using the original FSL device at the sensor data assessment.

Data on age, gender, postcode, diabetes duration, HbA1c, device start dates and insulin delivery modality were collected from electronic outpatient records. Participants were assigned an English index of multiple deprivation rank (IMD) [12] based upon their postcode [13]. These were then grouped by deprivation decile defined by their position in the ranks from the 32,844 small areas in England subdivided into ten equal groups: group 1 being the most socio-economically deprived, and 10 being the least deprived.

Analyses were performed using paired sample *t*-tests for normal variable distributions or Wilcoxon signed-rank tests for non-normal variable distributions. The relationships of demographic factors with sensor-based metrics were investigated using the Mann-Whitney *U* Test. Data are presented as mean ± SD or median (interquartile range). The hypothesis testing was ordered at the 0.05 level without any control for multiple testing. We completed analyses with SPSS (IBM software, Hampshire, UK, version 25). All *p* values are two-sided. We also compared the laboratory HbA1c before starting the sensor with the most recent sensor-based estimated HbA1c, also termed Glucose Management Indicator [14].

## 3. Results

We identified 789 adults with type 1 diabetes using either the Freestyle Libre (*n* = 591) or Dexcom systems (*n* = 198). Baseline characteristics of the study cohort are summarised by system type in Table 1. The majority of Dexcom users (63.1%), and half of the FSL users were treated with Continuous Subcutaneous Insulin Infusion (CSII), with the remainder treated with multiple daily injections. The median age of FSL users was 40 years with 22 years of T1DM diabetes, while the median age of Dexcom users was 38 years old with a duration of diabetes of 23 years.

### 3.1. HbA1c Changes

#### 3.1.1. Freestyle Libre Device

Pre and post paired HbA1c levels were available for 336/591 (56%) of the cohort. (Table 2). Overall HbA1c level improved from 61.0 (54.0, 71.0) to 57.0 (49.0, 65.8) within 1 year of starting the FSL device (*p* < 0.001). This improvement was highest in those with pre-sensor HbA1c values ≥69 mmol/mol (median change: −12 mmol/mol, *p* < 0.001) compared to those with pre-sensor HbA1c values between 59.0–68.9 mmol/mol (median change: −4 mmol/mol, *p* = 0.002). There was no improvement in HbA1c in those with pre-sensor HbA1c < 59.0 mmol/mol (*p* = 0.5).

#### 3.1.2. Dexcom Device

Pre and post paired HbA1c levels were available for 130/198 (65%) of the cohort (Table 2). Overall HbA1c level improved from 60.0 (50.0, 70.0) to 58.8 (50.3, 66.8) within 1 year of starting the Dexcom sensor (*p* < 0.001). Similar to the FSL device, Dexcom G6 users with baseline HbA1c levels of 59.0–68.9 mmol/mol (*p* = 0.001) and >69.0 mmol/mol (*p* = 0.01) showed statistically significant reductions in HbA1c level with no significant HbA1c change in those with baseline HbA1c values < 59.0 mmol/mol.

### 3.2. Sensor-Based Metrics

Key sensor-based metrics for the two sensors are shown in Table 3. Glucose Management Indicator (GMI) and other key sensor-based parameters were broadly similar between the two sensors. The median time spent in the hypoglycaemia range was low in users of both sensors. The time spent in the ‘high’ and ‘very high’ glucose ranges was also broadly comparable.

In this cohort, 23% of FSL users and 32 % of Dexcom users met the international target of achieving at least 70% of the time spent in the target glucose range of 3.9 to 10 mmol/L. Regarding targets for hypoglycaemia, 70% of Dexcom users and 58% of FSL users spent less than 4% of the time in the hypoglycaemia range (<4 mmol/L).

We also compared the laboratory HbA1c before starting the sensor with the most recent sensor based estimated HbA1c (also termed Glucose Management Indicator) for the whole cohort (*n* = 623). Median (IQR) laboratory pre-sensor HbA1c was 61 (53, 71), and median (IQR) GMI in the most recent three months was 57 (51, 64) with *p* < 0.001.

### 3.3. Demographic Predictors of Time Spent in the Target Glucose Range (3.9 to 10 mmol/L)

We compared time spent in the target glucose range in groups stratified by gender, age (≤30 vs. >30 years), Multiple Deprivation Index (1–5 vs. 6–10) and insulin delivery modality (CSII vs. MDI; Table 4).

Among FSL users, higher TIR was seen in males, in those with lower levels of socioeconomic deprivation and in older patients.

There was a trend for higher TIR in males and those with lower levels of deprivation for the Dexcom sensor, but these differences did not reach statistical significance. Importantly, there was no difference in TIR between insulin pump users and injection users regarding the Dexcom device, but there was a trend without statistical significance in FSL pump users to have slightly better TIR than MDI users.

## 4. Discussion

### 4.1. Main Findings

Our data show that under real-life conditions, the use of both FSL and Dexcom sensors is associated with significant improvements in HbA1c in those with baseline HbA1c values >59 mmol/mol. The magnitude of improvement was greater in those with higher baseline HbA1c values, particularly for FSL sensor users. In contrast, for the Dexcom sensor, broadly comparable improvements in HbA1c were noted between pre-sensor HbA1c levels 59.0 to 68.9 and ≥69.0 mmol/mol. The patient sensor usage was high, with more than 85% median sensor use for both sensors. In this cohort, the time spent in hypoglycaemia was generally low, with 65% of FSL users and 74% Dexcom users meeting the international target of time spent in hypoglycaemia (<3.9 mM) to be less than 4%. Our data also show the challenges of living with type 1 diabetes, with only 23% of FSL users and 32% of Dexcom users achieving the international target of spending 70% or more in the target glucose range 3.9 to 10 mmol/L. Further, in our cohort, males, people living in areas of lower deprivation and people over 30 years of age achieved more time in the target range with the FSL device.

### 4.2. Comparison with Other Studies—FSL Device

In the ABCD nationwide audit (*n* = 3182 with follow up data), FSL users demonstrated an HbA1c improvement of −5.2 mmol/mol after 7.5 months (baseline: 67.5 mmol/mol; follow-up: 62.3 mmol/mol). It is of note that our study pre-sensor HbA1c was lower than the ABCD follow-up HbA1c, which might explain why the overall improvement observed in our cohort was smaller than that observed in the ABCD nationwide audit. The magnitude of improvement of HbA1c in those with HbA1c > 69.0 mmol/mol was comparable with the ABCD nationwide audit around −12 mmol/mol. In addition, the median TIR in our study was 55%—much higher than the reported TIR of 43% in the ABCD audit [15].

A recent meta-analysis by Evans et al. evaluated FSL use in adults (*n* = 1023) and children (*n* = 447) for 12 months. The authors concluded that starting patients on Freestyle libre led to an HbA1c reduction of 0.56% (6.1 mmol/mol) for adults and 0.54% (5.9 mmol/mol) for children and adolescents, which was noted within 2 to 4 months of initiation and sustained for at least 12 months in adults [16].

In another study by Fokkert et al. from the Netherlands, FSL use (*n* = 1365) led to significant improvements in HbA1c at six months and 12 months with a difference of −4 mmol/mol for the whole cohort improving from 64.1 to 60.1 mmol/mol after 12 months (*p* < 0.001). In line with the current study, the authors found a more significant −9 mmol/mol improvement in those with a starting A1c > 70 mmol/mol [17].

### 4.3. Comparison with Other Studies—Dexcom Device

There have been many high-quality randomised controlled trials of the Dexcom device in a range of populations. These studies include DIAMOND [18], GOLD [19] studies in adults with type 1 diabetes, CITY [20] and MILLENNIALS [21] study in adolescents and young adults and WISDOM [22] study in older adults living with type 1 diabetes. These studies have consistently demonstrated HbA1c improvements between −0.4 (4.3 mmol/mol) to −0.8% (8.7 mmol/mol) using the Dexcom device. Further, the HypoDE study [23] demonstrated a reduction in biochemical and severe hypoglycaemia burden with Dexcom use.

Recent real-world publications from the USA have also explored patterns of CGM use and glycaemic outcomes from Dexcom. Linden et al. [24] report data from a large cohort of users who switched from the Dexcom G5 system to Dexcom G6 in December 2018. (*n* = 31,034). In this cohort, approximately 57.3 to 60.6% of glucose values were between 3.9 to 10 mmol/mol, which is comparable to our study with TIR 58.8%. Between 27 to 35% of people in this large cohort achieved the international target of >70% TIR. Again, this is in keeping with our study, with 32% of users meeting the above criteria.

In another real-world study, Akturk et al. [25] explored glucose data from USA-based Dexcom G6 users and assessed relationships with various system features, such as alert features, remote data sharing, software for retrospective data analysis and virtual assistant features. Individuals who used more of the alert and notification features had more favourable glycaemic outcomes. For example, those who used more than four features had a higher time spent in the target range than those who used less than three features (62.6% vs. 58.6%, *p* < 0.01).

### 4.4. Strengths and Limitations

Strengths of this study include a large sample size, use of average HbA1c values to assess outcomes and a high level of sensor data availability. To the best of our knowledge, it is also the first real-world study of Dexcom G6 device from the UK. Limitations include the real-world observational nature of our study with the lack of a comparator group. A small number of users in the FSL group were using the Freestyle libre 2 sensor (with the options of hypo and hyperglycaemic alarms at the time of sensor data analysis), but we are unable to quantify the precise number. Due to the COVID-19 pandemic since March 2020 the number of face-to-face visits and laboratory HbA1c measurements have been reduced. This decreased the % with paired laboratory HbA1c levels. However, we also looked at sensor-based HbA1c (GMI) levels in users with a high percentage of sensor use. We did not compare results between FSL and Dexcom users as these are two fundamentally different cohorts with different selection criteria. The Dexcom system is the only sensor currently funded in the NHS for people with high hypoglycaemia burden, impaired hypoglycaemia awareness, or severe fear of hypoglycaemia. Since such factors influence the ability to achieve near-normal glucose levels, it would have been incorrect to compare these two groups of patients directly. We did not collect information about any other adjunctive therapies apart from insulin, but we estimate only a very small percentage to be on such therapy. We also did not collect information about the number of out-patient clinic visits per participant but typically patients are reviewed at 6 to 12 monthly intervals in our centre.

## 5. Conclusions

In conclusion, in people with type 1 diabetes under routine clinical care in the UK, we report clinically significant HbA1c improvements in FSL and Dexcom users when the baseline HbA1c was >59 mmol/mol. In addition, the hypoglycaemia burden was low in our cohort with 65% of FSL users and 74% Dexcom users meeting the international target of time spent in hypoglycaemia (<3.9 mM) to be less than 4%. The HbA1c and TIR achieved using these devices are comparable to other published studies. Further, in our cohort, males, people living in areas of lower deprivation and people over 30 years of age achieved more time in the target range with the FSL device. Data from our cohort adds to the growing body of evidence supporting the FSL and Dexcom device in type 1 diabetes.

## Figures and Tables

**Table 1:** Baseline Characteristics.

Sensor Used	Freestyle Libre (*n* = 591)	Dexcom G6 (*n* = 198)
	Data (*n*/% or median (IQR))	
*n* (%)	591 (74.9)	198 (25.1)
Females	280 (47.4)	126 (63.6)
Age, years *	40 (30,51)	38 (30,51)
Diabetes duration, years *	22 (13, 32)	23 (15, 33)
Ethnicity, *n* (%)		
White	384 (64.9)	153 (77.3)
Black	20 (3.4)	3 (1.5)
Asian	33 (5.6)	9 (4.5)
Other	9 (1.5)	7 (3.5)
Not Specified	144 (24.4)	26 (13.1)
Diabetes therapy, *n* (%)		
CSII	292 (49.4)	125 (63.1)
MDI	292 (49.4)	57 (28.8)
Not Specified	7 (1.2)	16 (8.1)
Multiple Deprivation Index **		
1–5	323 (54.7)	102 (51.5)
6–10	264 (44.7)	95 (48)

Data are *n* (%) unless stated. * Median (IQR);.** based upon postcode. group 1 being the most socio-economically deprived, and 10 being the least deprived.

**Table 2:** HbA1c changes within one year of starting Freestyle Libre (Generation 1) and Dexcom (G5 and G6) systems in people with type 1 diabetes.

	Pre HbA1c	Post HbA1c	Change HbA1c (Pre-Post A1c)	*p* Value *
Freestyle Libre (Generation 1)				
All (*n* = 336)	61.0 (54.0, 71.0)	57.0 (49.0, 65.8)	3.5 (−3.0, 11.0)	<0.001
Baseline HbA1c (mmol/mol)				
<59.0 (*n* = 139)	52.0 (47.0, 56.0)	52.0 (47.0, 57.0)	0.0 (−5.0, 4.0)	0.503
59.0–68.9 (*n* = 95)	62.0 (60.5, 65.0)	59.0 (54.0, 66.0)	4.0 (−3.5, 9.0)	0.002
≥69.0 (*n* = 102)	76.0 (71.4, 89.0)	65.0 (53.4, 73.0)	12.3 (3.9, 25.0)	<0.001
Dexcom systems (G5/G6)				
All (*n* = 130)	60.0 (50.0, 70.0)	58.8 (50.3, 66.8)	2.5 (−2.5, 7.5)	0.002
Baseline HbA1c (mmol/mol)				
<59.0 (*n* = 56)	50.0 (43.9, 55.5)	49.5 (43.0, 54.0)	−0.8 (−2.9, 3.9)	0.938
59.0-68.9 (*n* = 36)	63.8 (61.5, 65.5)	59.0 (55.0, 62.6)	4.8 (0.4, 9.0)	0.001
≥69.0 (*n* = 38)	78.3 (71.4, 92.9)	72.3 (64.9, 92.4)	4.8 (−2.6, 10.9)	0.01

Data are median (IQR). * Within-person changes assessed by the Wilcoxon Signed Ranks Test.

**Table 3:** Summary of sensor-based metrics for people with type 1 diabetes using FSL and Dexcom G6 sensors.

Sensor Used	Freestyle Libre (*n* = 591)	Dexcom G6 (*n* = 177)
	Data (median (IQR))	
% Activity	85.0 (54.0, 97.0)	94.5 (85.7, 97.4)
Duration of sensor use (Months)	21.6 (10.1, 29.6)	24.4 (10.4, 38.1)
Average Glucose levels (mmol/L)	9.5 (8.2, 11.1)	9.4 (8.1, 10.8)
GMI (mmol/mol)	57.4 (51.3, 64.9)	56.9 (50.8, 63.5)
CV (%)	37.6 (33.7, 42.5)	36.1 (33.2, 40.0)
% In very low range (<3.0 mmol/L)	0.0 (0.0, 1.0)	0.3 (0.1, 1.0)
% In low range (3.0 to 3.8mmol/L)	3.0 (1.0, 5.0)	1.6 (0.7, 3.3)
% In target range (3.9 to 10.0 mmol/L)	55.0 (41.0, 68.0)	58.8 (42.7, 73.5)
% In high range (10.1 to 13.9 mmol/L)	25.0 (19.0, 30.0)	23.8 (17.4, 29.4)
% In very high range (>13.9 mmol/L)	12.0 (5.0, 25.0)	11.5 (3.95, 21.6)

GMI = Glucose Management Indicator. CV = Coefficient of Variation.

**Table 4:** Comparison of median time in range (3.9 to 10 mM) by gender, age, multiple deprivation index and diabetes therapy in people with type 1 diabetes.

	Freestyle Libre (*n* = 591)		Dexcom G6 (*n* = 177)	
	Median % time in range (IQR)	*p*-value *	Median % time in range (IQR)	*p*-value *
Gender				
Male	57 (42, 71)	0.017	64 (47, 76)	0.076
Female	53 (41, 66)		56 (42, 71)	
Multiple Deprivation Index				
1–5	52 (38, 66)	<0.0005	57 (37, 72)	0.096
6–10	59 (46, 70)		60 (47, 74)	
Diabetes Therapy				
CSII	58 (44, 68)	0.124	59 (47, 71)	0.613
MDI	53 (39, 68)		60 (39, 77)	
Age Group				
≤30	50 (36, 64)	0.003	56 (42, 73)	0.451
>30	56 (43, 70)		59 (43, 74)	

* Mann-Whitney *U* test.

## Supplementary Materials

The following are available online at https://www.mdpi.com/article/10.3390/bios11110457/s1. Table S1: NHS England Criteria for Freestyle Libre device.
