# Supplementary material for: Real-World Outcomes of Glucose Sensor Use in Type 1 Diabetes—Findings from a Large UK Centre
Source: Biosensors (Basel). 2021 Nov 15;11(11):457. doi: 10.3390/bios11110457 (PMC8615559; doi:10.3390/bios11110457)
Supplement: Supplementary file 1 [file biosensors-11-00457-s001.zip › biosensors-1446783-supplementary.pdf]

Supplementary Table S1:

NHS England Criteria for Freestyle Libre device:
Available at: https://www.england.nhs.uk/publication/flash-glucose-monitoring-national-arrangements-for-funding-of-relevant-diabetes-patients/
Accessed on 01 Oct 2021

1. People with Type 1 diabetes

   OR with any form of diabetes on hemodialysis and on insulin treatment

   who, in either of the above, are clinically indicated as requiring intensive monitoring >8 times daily, as demonstrated on a meter download/review over the past 3 months

   OR with diabetes associated with cystic fibrosis on insulin treatment

2. Pregnant women with Type 1 Diabetes - 12 months in total inclusive of post-delivery period.
3. People with Type 1 diabetes unable to routinely self-monitor blood glucose due to disability who require carers to support glucose monitoring and insulin management.
4. People with Type 1 diabetes for whom the specialist diabetes MDT determines have occupational (e.g. working in insufficiently hygienic conditions to safely facilitate finger-prick testing) or psychosocial circumstances that warrant a 6- month trial of Libre with appropriate adjunct support.
5. Previous self-funders of Flash Glucose Monitors with Type 1 diabetes where those with clinical responsibility for their diabetes care are satisfied that their clinical history suggests that they would have satisfied one or more of these criteria prior to them commencing use of Flash Glucose Monitoring had these criteria been in place prior to April 2019 AND has shown improvement in HbA1c since self- funding.
6. For those with Type 1 diabetes and recurrent severe hypoglycemia or impaired awareness of hypoglycemia, NICE suggests that Continuous Glucose Monitoring with an alarm is the standard. Other evidence-based alternatives with NICE guidance or NICE TA support are pump therapy, psychological support, structured education, islet transplantation and whole pancreas transplantation. However, if the person with diabetes and their clinician consider that a Flash Glucose Monitoring system would be more appropriate for the individual’s specific situation, then this can be considered.
7. People with Type 1 diabetes or insulin treated Type 2 diabetes who are living with a learning disability and recorded on their GP Learning Disability register.

NICE criteria for Real-time glucose monitor
Available at: https://www.nice.org.uk/guidance/ng17
Accessed on 01 Oct 2021

Consider real-time continuous glucose monitoring for adults with type 1 diabetes who are willing to commit to using it at least 70% of the time and to calibrate it as needed, and who have any of the following despite optimised insulin therapy and conventional blood glucose monitoring:

- More than 1 episode a year of severe hypoglycaemia with no obvious preventable cause.
- Complete loss of hypoglycaemia awareness.
- Frequent (more than 2 episodes a week) asymptomatic hypoglycaemia that is causing problems with daily activities.
- Extreme fear of hypoglycaemia.
- Hyperglycaemia (HbA1c level of 75 mmol/mol [9%] or higher) that persists despite testing at least 10 times a day
